# Navigated intramedullary nailing for patients with intertrochanteric hip fractures is cost-effective at high-volume hospitals in mainland China: A markov decision analysis

**DOI:** 10.3389/fsurg.2022.1048885

**Published:** 2023-01-16

**Authors:** Liang Tang, Xiaoke Yi, Ting Yuan, Hua Li, Cheng Xu

**Affiliations:** ^1^ Department of Orthopaedics, Hengyang Central Hospital, The Affiliated Hengyang Hospital of Southern Medical University, Hengyang, China; ^2^ Senior Department of Orthopaedics, The Fourth Medical Center of Chinese PLA General Hospital, Beijing, China; ^3^ National Clinical Research Center for Orthopedics, Sports Medicine and Rehabilitation, Beijing, China

**Keywords:** cost-effectiveness analysis, intertrochanteric fracture, intramedullary nailing, markov decision model, surgical volume

## Abstract

**Objective:**

Previous studies have reported that navigation systems can improve clinical outcomes of intramedullary nailing (IMN) for patients with intertrochanteric fractures. However, information is lacking regarding the relationship between the costs of navigated systems and clinical outcomes. The present research aimed to evaluate the cost-effectiveness of navigated IMN as compared with traditional freehand IMN for patients with intertrochanteric fractures.

**Methods:**

A Markov decision model with a 5-year time horizon was constructed to investigate the costs, clinical outcomes and incremental cost-effectiveness ratio (ICER) of navigated IMN for a 70-year-old patient with an intertrochanteric fracture in mainland China. The costs [Chinese Yuan (¥)], health utilities (quality-adjusted life-years, QALYs) and transition probabilities were obtained from published studies. The willingness-to-pay threshold for ICER was set at ¥1,40,000/QALY following the Chinese gross domestic product in 2020. Three institutional surgical volumes were used to determine the average navigation-related costs per patient: low volume (100 cases), medium volume (200 cases) and high volume (300 cases).

**Results:**

Institutes at which 300, 200 and 100 cases of navigated IMN were performed per year showed an ICER of ¥43,149/QALY, ¥76,132.5/QALY and ¥1,75,083/QALY, respectively. Navigated IMN would achieve cost-effectiveness at institutes with an annual volume of more than 125 cases.

**Conclusions:**

Our analysis demonstrated that the navigated IMN could be cost-effective for patients with inter-trochanteric fracture as compared to traditional freehand IMN. However, the cost-effectiveness was more likely to be achieved at institutes with a higher surgical volume.

## Introduction

Hip fractures are considered the most substantial public health problem faced by surgeons, health care systems and society with the ageing population (1). The economic burden of hip fractures is estimated to account for 72% of the total cost of all fractures (2), and the annual cost of hip fracture care in the United States will exceed 16 billion dollars by 2040 (3). In addition, hip fractures are associated with high mortality, which is reported to reach 27% in the first postoperative year (4).

More than half of hip fractures are intertrochanteric fractures, which are associated with older age and severe comorbidities (5), suggesting an enormous socio-economic health burden (6). This type of fracture is usually treated by internal fixation using intramedullary nailing (IMN) (7). However, the failure rate of IMN is suboptimal at 8% (8, 9). IMN failure is significantly related to the lag screw position, which is commonly assessed by the tip-apex distance (TAD). Several studies have recommended a TAD threshold of less than 20 mm for the lag screw in IMN to ensure secure fixation and avoid complications (10, 11). Nevertheless, this target is quite challenging for surgeons under fluoroscopic guidance.

As the implantation of an accurate and precise lag screw is essential for IMN, navigation systems have been developed and employed in this procedure to assist surgeons. It is reported that the systems enable surgeons to facilitate screw implantation. Previous studies have shown that the navigation systems have better accuracy of screw insertion than the traditional freehand method (12, 13). However, a natural disadvantage of this new technique is the high economic expenditure. The cost of computer-navigated devices in clinical application can reach £7,00,000 (14). It is therefore important to investigate whether the incremental costs incurred by the systems can be equated with the improved outcomes. To our knowledge, information is lacking regarding the relationship between the cost of navigated systems and clinical outcomes in intertrochanteric fractures. Thus, we aimed to evaluate the cost-effectiveness of navigated IMN as compared with traditional freehand IMN for patients with intertrochanteric fractures.

## Materials and methods

### Overview of the study

The present study was conducted according to the guideline of the Panel on Cost-Effectiveness Analysis in Health and Medicine (15). A pay perspective was employed to evaluate the cost and effectiveness. The unit of cost was 2020 Chinese Yuan (CNY, ¥) and the unit of effectiveness (utility) was the quality-adjusted life-year (QALY). The assessment of cost-effectiveness was based on the calculation of incremental cost-effectiveness ratio (ICER) and the willingness-to-pay (WTP) threshold. The ICER, which is the difference in costs between two procedures divided by the difference in utility, can be expressed as Δ Costs/Δ Utility. The WTP threshold represents how many economic expenditures a patient will pay to gain an extra QALY (14). If the ICER of a procedure was below the WTP threshold, the procedure was considered cost-effective. The World Health Organization has recommended that the WTP threshold be set at 1–3 times the gross domestic product per QALY (16). In our analysis, the WTP threshold was set at ¥1,40,000 following the Chinese gross domestic product (¥71,489) in 2020 (17)^1^. A theoretical 70-year-old patient with an intertrochanteric fracture was set for the reference case analysis. In order to calculate the future parameters during the time horizon given the economic growth, a discount rate of 3% was used for the cost and QALYs (18). It should be noted that though the discount rate could adjust and simplify the model, this simulation might not reflect the changes in operations, health utility and cost over the time horizon comprehensively.

### Model design

**Figure 1:**
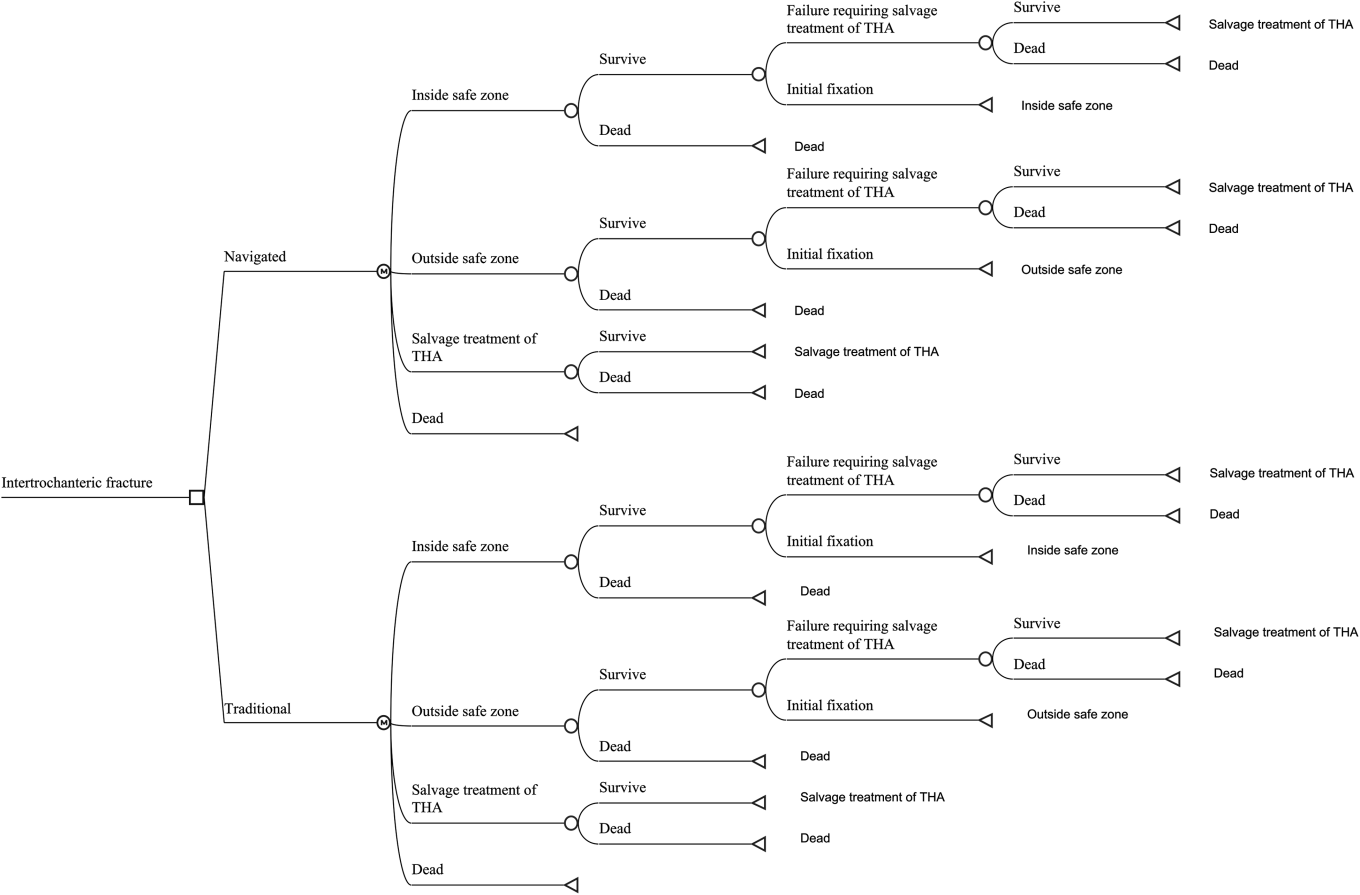
The markov model for patients with intertrochanteric hip fractures. Each patient received in-tramedullary nailing (IMN), either with navigated or traditional assistance. The patient received the IMN procedure and the lag screw was possibly inside or outside the safe zone. If a patient survived the perioperative period, that patient would stay in the status of successful initial fixation with the lag screw inside or outside the safe zone, experience IMN failure requiring salvage treatment of total hip arthroplasty (THA) or die from other causes.

Only the data from the vendor or published studies were used in the present research. We built a Markov decision model using the decision analysis software (TreeAge Pro 2019; TreeAge Software, Williamstown, MA) for two competing strategies: navigated IMN or traditional freehand IMN. The navigation system, analyzed in this study, was the TiRobot navigation system (TINAVI Medical Technologies, Beijing, China). The type of IMN was the Gamma3 trochanteric nail (Gamma3, Stryker, Mahwah, NJ, USA), as most of the published studies regarding the navigated IMN applied this type of implant. The value of each model parameter was listed in Table 1 and the parameters were described individually below. As previous publications comparing navigated IMN with traditional IMN had a short follow-up period (13, 25–27, 31–33), we used a 5-year time horizon for the present analysis. The model considered four states, which were categorized as follows: (1) successful IMN with the lag screw inside the safe zone, (2) successful IMN with the lag screw outside the safe zone, (3) salvage treatment of total hip arthroplasty (THA) after failed IMN and (4) death. The safe zone was defined as a lag screw TAD of <20 mm. At first, the patient would receive IMN with or without navigation. After the procedure, the patient would either have a successfully initial fixation of fracture, encounter the failure of fixation requiring salvage treatment of THA, or die of other causes each year. The patient was also at risk of perioperative death before the procedure of IMN and salvage treatment (Figure 1).

**Table 1:** Values of parameters used in the markov decision model.

Parameter	Value (reference case)	Reference
***Probability***		
Perioperative mortality for IMN	6%	(19, 20)
Perioperative mortality for THA	6%	(19, 20)
Mortality of IMN during the first postoperative year	20%	(19)
Mortality after the first year	Age-specific mortality	(21)
Annual failure probability of IMN with TAD < 20 mm (inside the safe zone)	2.7%	(22)
Annual failure probability of IMN with TAD > 20 mm (outside the safe zone)	7.5%	(11, 23, 24)
Probability of navigated IMN inside the safe zone	95.6%	(13, 25, 26)
Probability of traditional IMN inside the safe zone	74.0%	(13, 25, 26)
***Utility (QALY)***		
Navigated IMN	0.82	(27)
Traditional IMN	0.79	(27)
Salvage treatment of THA	0.76	(19, 20)
Disutility for salvage treatment	−0.15	(19)
***Cost (¥)***		
IMN procedure	54,000	(28)
Navigation system	70,00,000	Vendor (TiRobot)
Maintenance fees	5% of purchase costs	Vendor (TiRobot)
Salvage treatment	1,08,000	(29, 30)
***Other***		
WTP threshold	1,40,000	(17)
Discount rate	3%	(18)

IMN, intramedullary nailing; THA, total hip arthroplasty; TAD, tip-apex distance; QALY, quality-adjusted life-year; WTP, willingness-to-pay.

### Assumptions

We made a few assumptions were made in constructing the model: (1) the probability of perioperative death and the mortality of other causes was the same in both navigated and traditional IMN; (2) the failure probability of IMN was the same in both navigated and traditional IMN; (3) salvage treatment of THA would not fail during the time horizon.

### Probabilities

We set the perioperative mortality of IMN and THA at 6%, according to published data (19, 20). The mortality of IMN in the initial year postoperatively was set at 20% (19). After the first postoperative year of IMN, the mortality was assumed to return to the age-specific mortality reported by the China Populations Census 2020 (19, 21)^2^. The mortality after THA was also assumed to be the same as age-specific mortality.

According to an analysis of the Norwegian hip fracture register of 17,341 patients, the annual failure rate of IMN placed inside the safe zone was set at 2.7% (22). There were three available studies reporting the odds ratios of TAD (TAD > 20 mm VS TAD < 20 mm) with regard to the IMN failure (11, 23, 24). The pooled odds ratio was calculated to be 2.94 by the meta-analysis. Thus, we set the annual failure rate of IMN placed outside the safe zone at 7.5% using the above data and the method by Zhang et al. (34). Three studies compared the proportion of TAD < 20 mm in navigated IMN with that in traditional IMN, and the pooled results showed that navigated IMN could achieve 95.6% of safe IMN compared to 74.0% in traditional IMN (13, 25, 26). The two aforementioned rates were used when assigning the IMN inside or outside the safe zone.

### Health utility (effectiveness)

There is no concrete value for the QALY of navigated IMN in intertrochanteric fractures. Only the study by Lan et al. has reported the comparison of Harris Hip Score between patients receiving the navigated IMN and those receiving the traditional IMN. The HHS was 86.7 in the navigated group and 82.7 in the traditional group (27). Therefore, we used the method of Shearer et al. to convert the HHS into corresponding QALYs by linear interpolation (35). Based on the transformation, the health utility was set at 0.82 QALYs for the navigated IMN and 0.79 QALYs for the traditional IMN. These values were within the range of the mean utilities reported by other studies of intertrochanteric fractures (36, 37). The salvage treatment of THA was assigned with a health utility of 0.6 QALYs according to previous studies (19, 20). A disutility of −0.15 (QALY loss) was assigned to the salvage procedure.

### Costs

The cost of the IMN procedure in mainland China was set at ¥54,000 according to a national cost survey of 73 tertiary hospitals (28). The cost of the salvage THA (¥1,08,000) was set as twice the cost of the primary IMN according to previous studies (29, 30). The purchase and maintenance cost of navigation system were obtained from the vendor and integrated into the model. Cost per patient receiving the navigated IMN would be influenced by the surgical volumes. The base surgical volume of navigated IMN was set at 200 cases/year, low surgical volume at 100 cases/year and high surgical volume at 300 cases/year (38–41).

### Analysis

We first performed the analysis for the reference case. Then one-way and two-way deterministic sensitivity analyses were conducted. In addition, the parameters related to the navigation system, including the purchase cost, the utility of navigated IMN and the probability of safe navigated IMN, were estimated in each surgical volume to calculate the corresponding thresholds.

We used the Monte Carlo simulation to perform the probabilistic sensitivity analysis to determine the overall effect of uncertainty parameters (Table 2). The distribution of each variable was determined by the mean and the standard deviation (if available or set as 10% of the mean). The ICER was calculated using a simulation for 1,000 samples. The cost-effectiveness acceptability curve was used to determine the proportion of samples who had an ICER below the given WTP threshold.

**Table 2:** Parameters for probabilistic sensitivity analysis with monte carlo simulation.

Parameter	Distribution	*α*	*β*	Mean	SD
Annual failure probability of IMN with TAD < 20 mm (inside the safe zone)	Beta	97.27	3505.43	2.7%	0.27%
Annual failure probability of IMN with TAD > 20 mm (outside the safe zone)	Beta	92.43	1139.91	7.5%	0.75%
Probability of navigated IMN inside the safe zone	Beta	3.44	0.16	95.6%	9.56%
Probability of traditional IMN inside the safe zone	Beta	25.26	8.88	74.0%	7.40%
Utility of navigated IMN (QALY)	Beta	17.18	3.77	0.82	0.082
Utility of traditional IMN (QALY)	Beta	20.21	5.37	0.79	0.079
Utility of THA (QALY)	Beta	3999.4	2666.3	0.6	0.06
Costs of IMN procedure (¥)	Gamma	2.13	0.000039	54,000	37,000
Costs of THA (¥)	Gamma	100	0.00093	10,8000	10,800

SD, standard deviation; IMN, intramedullary nailing; TAD, tip-apex distance; QALY, quality-adjusted life-year; THA, total hip arthroplasty.

## Results

### Reference case

For the reference case, the navigated IMN had a cumulative quality of life of 3.32 QALYs and a total cost of ¥74,963. The state rate for salvage procedures was 8.5%. As for the traditional IMN, this strategy had a cumulative quality of life of 3.18 QALYs and a total cost of ¥64,290. The state rate for salvage procedures was 11.1%. Thus, the navigated IMN yielded an ICER of ¥76,132.5/QALY and a reduction of salvage procedure by 23.4% compared with the traditional IMN.

### Sensitivity analysis

**Figure 2:**
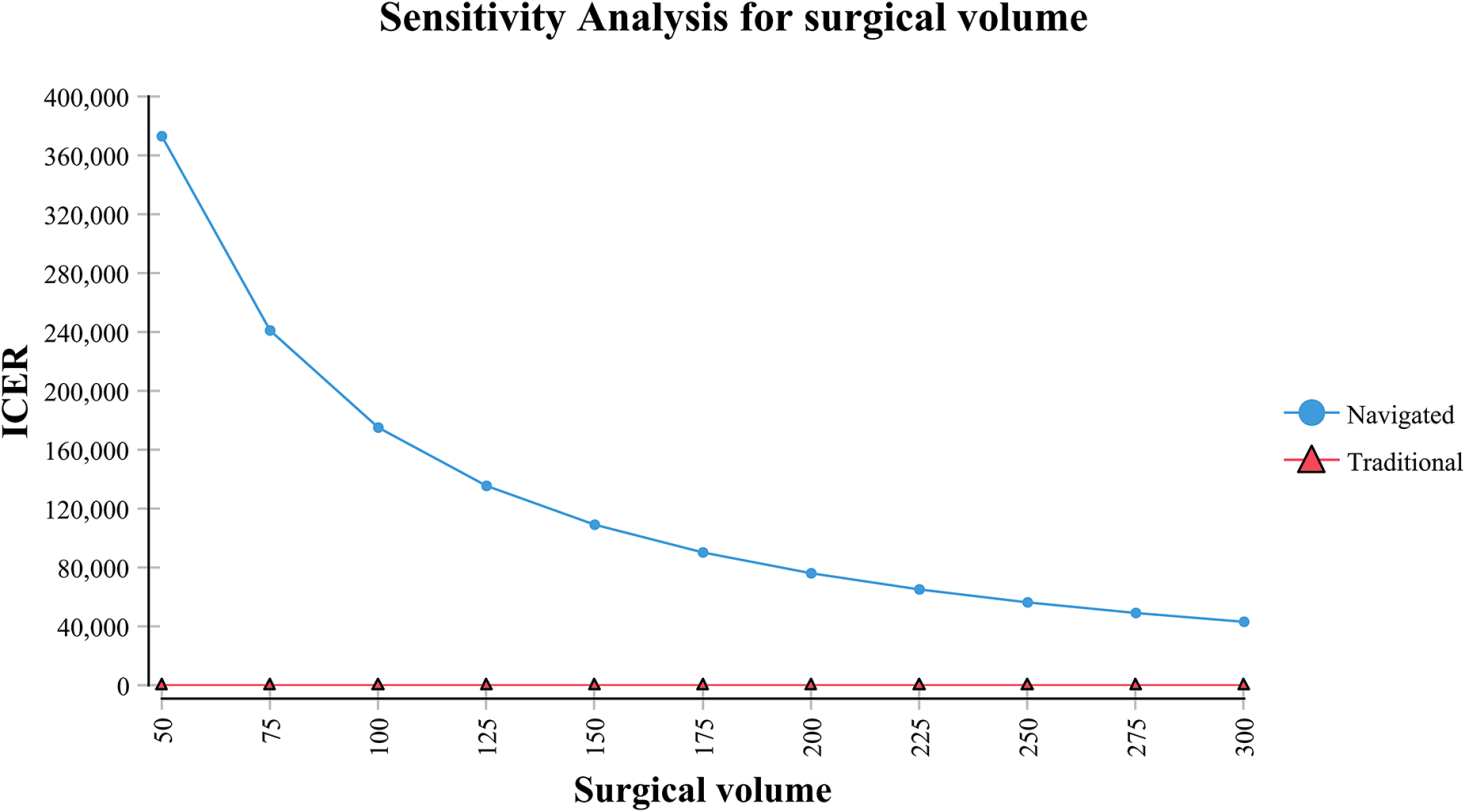
Sensitivity analysis reflected an inverse relationship between annual surgical volume of navigated surgery and incremental cost-effectiveness ratio (ICER).

In the scenarios with different surgical volumes, the navigated IMN was conditionally cost-effective (Table 3). It should be noted that at a low-volume institute, the navigated IMN could not be cost-effective even if all the IMN were placed inside the safe zone. An inverse relationship between volume and ICER of navigated IMN was observed (Figure 2). The navigated IMN showed cost-effectiveness at institutes with an annual volume of more than 125 cases. At a low-volume institute, the ICER of navigated IMN was ¥1,75,083/QALY. While at a high-volume institute, the ICER was ¥43,149/QALY.

**Table 3:** Thresholds for parameters related to the cost-efficiency of navigation in different volumes.

Parameter	Low volume (100 cases/year)	Medium volume (200 cases/year)	High volume (300 cases/year)
Probability of safe navigated IMN	No values	>67.3%	>53.1%
Utility of navigation (QALY)	>0.83	>0.80	>0.79
Costs of navigation system (¥)	<57,59,057	<1,15,18,114	<1,26,24,617

IMN, intramedullary nailing; QALY, quality-adjusted life-year.

Among other parameters that were not directly related to the navigation system, three (probability of safe traditional IMN, failure probability of safe IMN and utility of THA) showed a positive relationship with the ICER of navigated IMN, which indicated that the increase of these values would compromise the cost-effectiveness of the navigation system. Two parameters (cost of IMN procedure and failure probability of unsafe IMN) showed a negative relationship with the ICER of navigated IMN. The disutility of salvage procedure and the preoperative mortality seemed to be inconsequential (Supplementary Figure S1).

**Figure 3:**
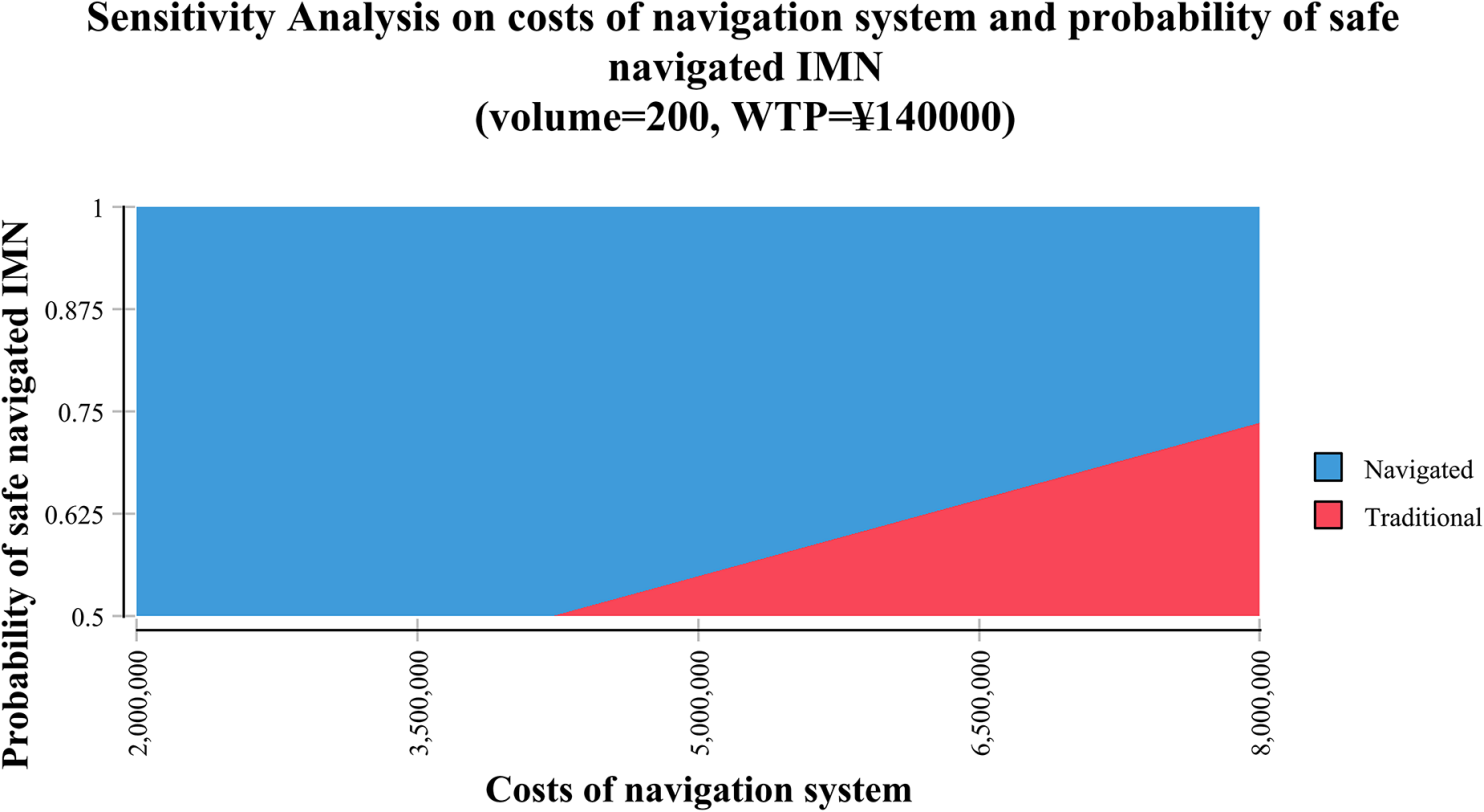
Two-way sensitivity analysis demonstrated the relationship between the cost of the navigation system and the probability of navigated intramedullary nailing (IMN) with the lag screw inside the safe zone. The blue area indicated the profiles for which the navigated IMN was more cost-effective (the cost was below the willingness-to-pay [WTP] threshold of ¥140,000 per quality-adjusted life-year [QALY]) in institutes with a volume of 200 cases per year. The red area indicated that the profiles for which the traditional IMN was more cost-effective in the same settings. CAOS: computer-assisted orthopaedic system; IV: Inverse variance method; CI: confidence intervals; df: degrees of freedom.

Two-way sensitivity analysis represented that the navigated IMN could be more possibly cost-effective when the cost of the navigation system was lower and the probability of safe IMN was higher (Figure 3).

**Figure 4:**
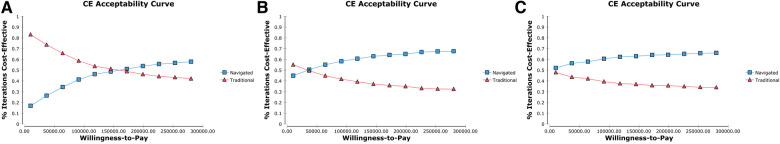
The cost-effectiveness (CE) acceptability curves depicted the relationship between the willing-ness-to-pay (WTP) threshold (Chinese Yuan per quality-adjusted life-year) on the X axis and the proportion of simulations in monte carlo simulation that had an incremental cost-effectiveness ratio below the given WTP thresholds. (**A–C**) Indicated the simulations in institutes at which 300, 200 and 100 cases of navigated surgery, respectively.

The probabilistic sensitivity analysis by Monte Carlo simulation demonstrated an ICER of navigated IMN as ¥1,67,401/QALY, ¥69,066/QALY and ¥35,327/QALY at a low-volume, medium-volume and high-volume institute, respectively. The cost-effectiveness acceptability curves showed that 48.4% of simulations had an ICER lower than the WTP threshold of ¥1,40,000 at low-volume institutes, 60.4% at medium-volume institutes and 63.3% at high-volume institutes (Figure 4).

## Discussion

To our knowledge, there has been no economic analysis estimating the cost-effectiveness of a navigation system for the IMN procedure. Our research is the first cost-effectiveness analysis to investigate the cost-efficiency of the navigated IMN from a payer perspective based on data from mainland China. We found that the navigated IMN could be cost-effective in hospitals with medium or high surgical volumes. In hospitals with low surgical volumes, on the other hand, the purchase burden added by the system might not be equivalent to the additional gain in patient QALYs.

Our model showed that the cost-effectiveness of navigated IMN could be achieved with a volume of at least 125 cases per year. The higher the surgical volume, the lower the ICER of navigated IMN could be, indicating that this new-generation device was more likely to be cost-effective. Our Monte Carlo simulation also reflected that as the surgical volumes increased, the proportion of samples with an ICER below the WTP threshold also increased. These results were similar to those of studies examining the cost-effectiveness of navigation systems in other orthopaedic disciplines. Slover et al. used a Markov model to evaluate the cost-effectiveness of navigation in total knee arthroplasty. They found that the annual reduction in failure rate for navigated surgeries at low-volume institutes must be 13% for the navigation system to be cost-effective, while the reduction at medium-volume institutes was required to be 2.5% (42). Another study found that the navigated unicompartmental knee arthroplasty could only achieve cost-effectiveness unless the annual case volume exceeded 94 cases (43). Dea et al. reported that the navigated spinal surgery could be cost-saving in centers performing 254 surgeries per year (44). Generally, the average costs per patient added by navigation systems could be offset by higher case volumes.

Several researches have demonstrated that the lower surgical volumes were related to higher rates of complications and failures in hip fracture procedures and other orthopaedic surgeries (45–47). In IMN, experienced surgeons were able to insert screws more precisely than inexperienced surgeons (48). Therefore, the navigated system could allow providers with small surgical volumes to improve the clinical outcomes. Our findings highlighted a critical dilemma for low-volume institutes where the improved outcomes were not equated with the increased costs. The threshold analysis revealed that a 17.7% reduction in the price of the navigation system (from ¥70,00,000 to ¥57,59,057) was required for the system to be considered cost-effective in a center performing 100 cases per year. The current model could be used as a guide for hospital administrators to evaluate the economic investment of navigation systems and to negotiate the price of systems with vendors. Slover et al. recommended several non-purchase options, such as leasing or rental agreements or cost-sharing strategies for low-volume institutes to reduce the cost per case of employing this new technology (42). Another factor that could influence the balance between the surgical volume and the price of system was the ability of navigation systems to implant a safe IMN. Future studies on the development of navigation systems are appealed in this direction.

This study had some limitations. First, the model parameter values were obtained from published sources rather than from prospective collections. Due to the paucity of data, patients' QALYs after the interventions were converted from HHS based on published methods. However, the calculation of QALY was generally derived from patient-reported outcome scores such as EuroQol five dimensions questionnaire. Therefore, we performed a sensitivity analysis to explore the impact of parameters on the cost-effectiveness. It should be noted that the model results were sensitive to several parameters. Second, nearly all current studies focusing on the navigated IMN utilized the Gamma3 nail, which is of a single lag screw. Our study was based on these data. However, there are several other widely-accepted implants for the IMN procedures, such as Proximal Femoral Nail Anti-rotation (PFNA, Synthes, Oberdorf, Switzerland) and InterTAN (Smith & Nephew, Memphis, TN, USA) with twin lag screws. Our results could not represent all the types of IMN procedures and should be interpreted carefully. Third, we have simplified the calculation of the cost of navigation system in our model by integrating the purchase and maintenance costs, which might actually neglect the service life of the system itself and the running cost when using a new technique. We believe that the consideration of the system longevity is not compulsory since we have used a relatively short time horizon of 5 years. However, with a longer time horizon, such as life expectancy, the service life of system should be a concern. Fourth, as few studies have reported the 5-year mortality of IMN or intertrochanteric fractures, we adopted the method by Swart et al. to simplify the model by elevating the mortality of IMN in the first postoperative year (19). We assumed that after the first year of IMN, the mortality would return to age-specific mortality. However, a higher mortality after IMN would also influence the cost-efficiency of the navigation system (Supplementary Figure S2). Thus, our method of simplification might lead to bias. Fifth, THA was set as the only salvage treatment for failure after primary IMN, as it is the most common therapy after failed internal fixation in patients with hip fractures. Therefore, other salvage treatments such as re-IMN or conservative treatment were not reflected in the model. Sixth, we assumed that the salvage THA would not fail during the 5-year time horizon, based on a 7-year follow-up study that reported a 100% survival rate of THA in 107 cases after a failed IMN (49). Sixth, the Chinese monetary system might jeopardize the generalizability of the results.

## Conclusions

Our early economic analysis demonstrated that the navigated IMN could be cost-effective in patients with intertrochanteric fractures as compared to traditional freehand IMN. However, the cost-effectiveness was more likely to be achieved at institutes with a higher surgical volume.

## Data Availability

The original contributions presented in the study are included in the article/**Supplementary Material**, further inquiries can be directed to the corresponding author/s.

## Footnotes

^1^ http://www.stats.gov.cn/tjsj/ndsj/2021/indexch.htm

^2^ http://www.stats.gov.cn/tjsj/pcsj/rkpc/7rp/indexch.htm
