# Supplementary material for: Navigated intramedullary nailing for patients with intertrochanteric hip fractures is cost-effective at high-volume hospitals in mainland China: A markov decision analysis
Source: Front Surg. 2023 Jan 16;9:1048885. doi: 10.3389/fsurg.2022.1048885 (PMC9885142; doi:10.3389/fsurg.2022.1048885)
Supplement: Supplementary file 1 [file Datasheet1.pdf]

Supplementary Material

Navigated Intramedullary Nailing for Patients with Intertrochanteric Hip Fractures is Cost-Effective at High-Volume Hospitals in Mainland China: a Markov Decision Analysis

Liang Tang*, Xiaoke Yi, Ting Yuan, Hua Li*, Cheng Xu*

*** Correspondence:** Liang Tang: tangliangorthop@163.com; Hua Li: leewahpkuhsc@outlook.com; Cheng Xu: xuchengngh@163.com

1 Supplementary Figures

Supplement Figure 1.

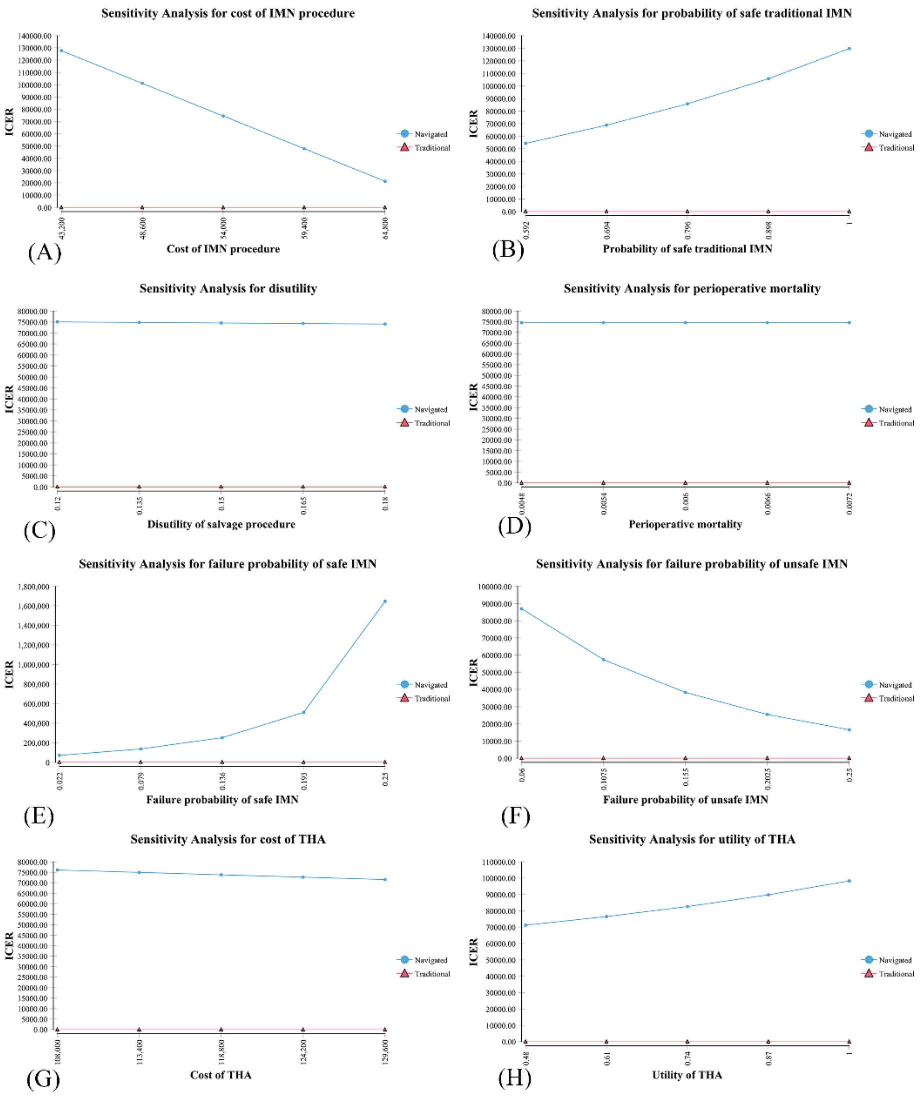

Supplement Figure 1. Sensitivity analysis for other parameters that are not directly related to the navigation systems. The blue line indicated the incremental cost-effectiveness ratio (ICER) of navigated intramedullary nailing (IMN) and the red line indicated the ICER of traditional IMN. (A) relationship between cost of IMN procedure and ICER; (B) relationship between the probability of traditional safe IMN and ICER; (C) relationship between the disutility of salvage total hip arthroplasty (THA) and ICER; (D) relationship between the perioperative mortality and ICER; (E) relationship between the failure probability of safe IMN and ICER; (F) relationship between the failure probability of unsafe IMN and ICER; (G) relationship between the cost of THA and ICER and (H) relationship between the utility of THA and ICER.

Supplement Figure 2.

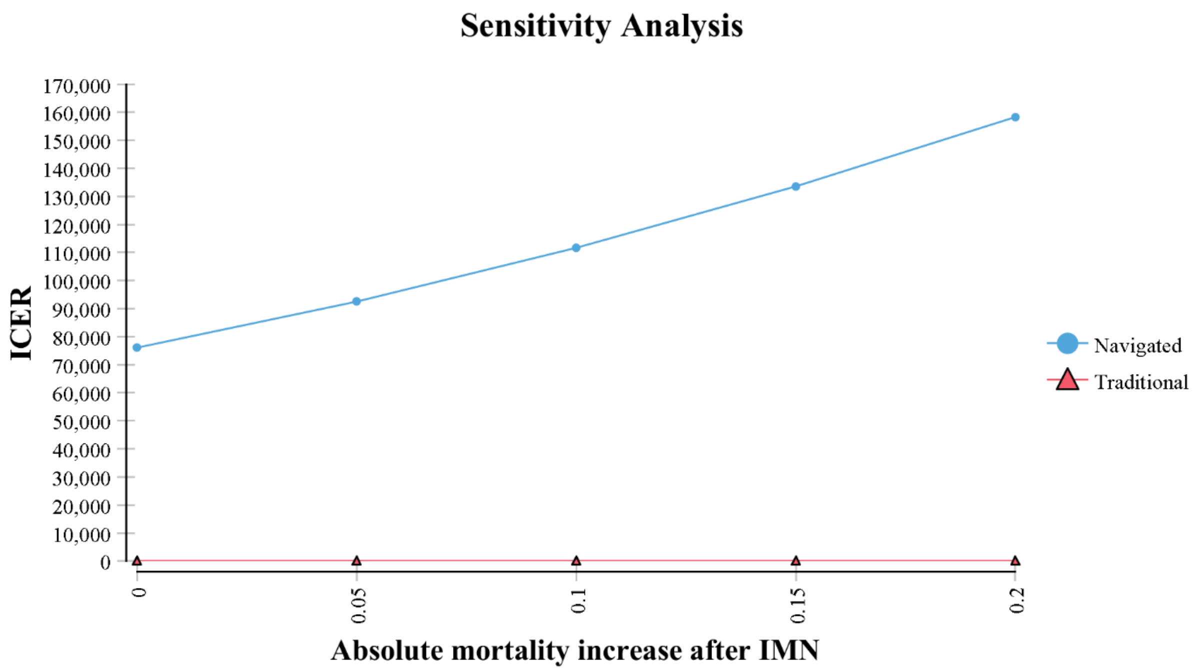

Supplement Figure 2. Sensitivity analysis reflected a positive relationship between absolute mortality increase after IMN in each year and incremental cost-effectiveness ratio (ICER) of navigation system
